# gff2sequence, a new user friendly tool for the generation of genomic sequences

**DOI:** 10.1186/1756-0381-6-15

**Published:** 2013-09-11

**Authors:** Salvatore Camiolo, Andrea Porceddu

**Affiliations:** 1 Dipartimento di Agraria, Università degli Studi di Sassari, Sassari 07100, Italy

**Keywords:** Gene annotation, General feature format, Sequence quality

## Abstract

**Background:**

General Feature Format (GFF) files are used to store genome features such as genes, exons, introns, primary transcripts etc. Although many software packages (i.e. *ab initio* gene prediction programs) can annotate features by using such a standard, a small number of tools have been developed to extract the corresponding sequence information from the original genome. However the present tools do not execute either a quality control or a customizable filter of the annotated features is available.

**Findings:**

gff2sequence is a program that extracts nucleotide/protein sequences from a genomic multifasta by using the information provided by a general feature format file. While a graphical user interface makes this software very easy to use, a C++ algorithm allows high performance together with low hardware demand. The software also allows the extraction of the genic portions such as the untranslated and the coding sequences. Moreover a highly customizable quality control pipeline can be used to deal with anomalous splicing sites, incorrect open reading frames and not canonical characters within the retrieved sequences.

**Conclusions:**

gff2sequence is a user friendly program that allows the generation of highly customizable sequence datasets by processing a general feature format file. The presence of a wide range of quality filters makes this tool also suitable for refining the *ab initio* gene predictions.

## Background

Advent of next generation sequencing, together with the organization of several genome projects, made sequencing the genome an affordable task for many organisms. Many gene prediction programs allow the identification of genes within a new genome [1] and the General Feature Format (GFF, proposed by Durbin and Haussler, http://www.sanger.ac.uk/software/gff/) is often chosen for storing the resulting data [2]. GFF format reports the genomic features in single-line records where information such as type and position are provided. Several tools have already been developed in order to deal with GFF files (refer also to the above URL). Programs such as BEDtools [3], readseq [4] and gff-ex (http://bioinfo.icgeb.res.in/gff/) can perform this task although their usage requires previous command line interface experience. Galaxy [5-7] is an easy to use alternative featuring a graphical user interface which is available as a stand alone version, as well as a web application. Although extremely versatile these programs have limitation in dealing with annotation data. As an example, the gene regions are not straightforwardly reconstructed and instead introns and exons sequences are generated. Enboss [8], together with its graphical user interface (GUI) version Jemboss [9], can also manage annotation files and convert them in a large number of formats. However all these software lack a downstream quality control of the output data (e.g. presence of anomalous characters within the nucleotide sequences, presence of canonical splicing sites in introns, etc.).

Additionally, dealing with annotation data may represent a non trivial task due to the heterogeneity underlying the way a GFF file can be written. Indeed the association between initial and final positions of a feature and its direction (5’ -> 3’ or *viceversa*), the presence of several splicing variants for the same gene, the possible occurrence of overlapping features should all be considered. This may require an intense scripting effort which can rarely be made by a user with basic informatics skills.

Here we present gff2sequence an open-source program which allows the extraction of gene features from an annotation file while controlling for several quality filters and maintaining a user friendly graphical environment.

### Implementation

C++ was used in order to implement the main algorithm of gff2sequence as well as for its graphic user interface which relies on the Qt-project library (qt-project.org).

## Results and discussion

**Figure 1:**
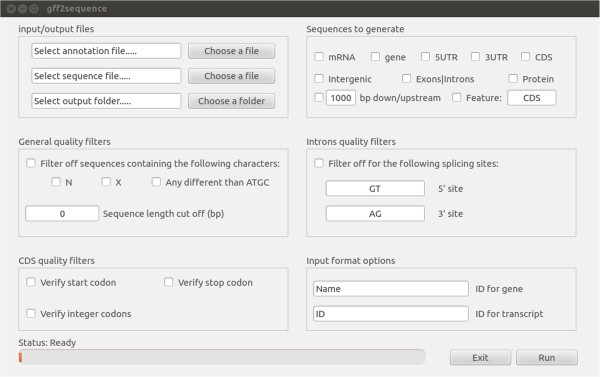
gff2sequence graphic user interface.

gff2sequence (Figure 1) takes in input a GFF (or GTF) annotation file and the relevant multifasta genome information and generates the nucleotide sequences of many genic and intergenic features (e.g. untranslated regions, coding sequences, proteins, introns, exons, genes, transcripts and down/upstream sequences). While the software was designed to work with gene annotation data it can also be used to extract generic features from any multifasta nucleotide sequence (see documentation for details).

Many parameters can be set in order to filter the output sequences. A general quality control can be used for selecting only sequences with no special characters (e.g. N for incomplete assembly, X for masking, etc.), or exceeding a user defined length. Coding sequences can be tested for the presence of a proper start codon (ATG), for the occurrence of a canonical stop signals (e.g. TGA, TAA or TAG), and for the presence of full codons (e.g. the total number of nucleotides is divisible by 3). Such features are automatically selected when the CDS translation is performed (standard genetic code is used to perform this task). Finally introns may be filtered by specifying the splicing signals.

gff2sequence generates three output files when the intergenic sequence information are gathered: (a) a multifasta formatted file reporting the nucleotide sequences, (b) a list of gene couples that are adjacent (e.g. with no intergenic sequences between them) and (c) a list of gene couples that are overlapping (e.g. genes which partially share a portion of DNA).

The software was tested on gff files that were available from a number of curators and performed smoothly for the following species (see Supplementary file “inputFileTested.pdf” for a complete list of the used URL): *Arabidopsis thaliana*[10], *Vitis vinifera*[11], (Phytozome [12]), *Solanum lycopersicum* (Sol Genomics Network at www. http://solgenomics.net/[13]), *Oryza sativa*[14] (Rice genome annotation project at http://rice.plantbiology.msu.edu/), *Zea mays*[15] (http://www.maizesequence.org/).

The main algorithm allows fast computations without being too demanding on hardware. As a way of example, full analysis (e.g. quality filters were all selected) of the large *Zea mays* genome (around 2500 Mbp) was performed in 11 minutes and required between 2.5 and 3 Gigabytes of memory on an Intel® Core™ i5 CPU M 430 working at 2.27 GHz. The presence of 2920 anomalous coding sequences and 928 overlapping genes emerged from such analysis.

## Conclusions

We believe gff2sequence may represent a valuable and easy to use alternative for the generation of a customized sequence dataset from general feature formatted file. Moreover identification of anomalous coding sequences, overlapping genes or non-canonical splicing sites may help in refining the automatic gene predictions.

### Availability and requirements

**Project name:** gff2sequence (version 0.1)

**Project home page:**http://sourceforge.net/projects/gff2sequence/

**Operating system:** Linux 64-bit

**Programming language:** C++

**Other requirements:** Qt library installed

**License:** GNU GPL

**Long term support:** Software support will be given for at least one year after release. Any bug will be analyzed and the software corrected. Each bug correction and/or software improvement will be followed by a new version release.

## Competing interests

Both authors declare that they have no competing interests.

## Authors’ contributions

SC was involved in the design and realization of the software. AP contributed to the project conception and participated to draft the manuscript. Both authors read and approved the final manuscript.
